# Medicare part D prescribing for direct oral anticoagulants in the United States: Cost, use and the “rubber effect”

**DOI:** 10.1371/journal.pone.0198674

**Published:** 2018-06-07

**Authors:** Panayiotis D. Ziakas, Irene S. Kourbeti, Loukia S. Poulou, Georgios S. Vlachogeorgos, Eleftherios Mylonakis

**Affiliations:** 1 Department of Medicine, Warren Alpert Medical School of Brown University, Providence, Rhode Island, United States of America; 2 First Department of Internal Medicine, Medical School, University of Athens, Greece; 3 Division of Medical Imaging & Interventional Radiology, “IASO” Maternity Clinic, Athens, Greece; 4 Iatriki Frontida Medical Center, Chalkida, Greece; 5 Warren Alpert Medical School of Brown University, Providence, Rhode Island, United States of America; Inselspital Universitatsspital Bern, SWITZERLAND

## Abstract

**Introduction:**

Direct oral anticoagulants (DOAC) have gained an increased share over warfarin for prevention and treatment of thromboembolic disease. We studied DOAC adoption across providers and medical specialties.

**Methods:**

Retrospective, cross-sectional analysis of Medicare Part D public use files (PUF), 2013 to 2015. We summarized prescription data for claims and drug payment, stratified by drug class, specialty and calendar year. We treated DOAC claims as a count outcome and explored patterns of expansion across prescribers via a truncated negative binomial regression. We described dispersion and spread in DOAC prescribing, across hospital referral regions (HRRs), including the p90/p10 ratios, and the median absolute deviation from the median.

**Results:**

In 2015 part D PUF, oral anticoagulant claims have climbed to approximately 24.4 million with a payment cost of approximately $3.3 billion. DOAC claims comprised 31.0% of oral anticoagulant claims, showing a relative increase of approximately 127% compared to 2013. The upper decile of prescribers accounted for half of the oral anticoagulant prescriptions and the resulting cost. The median cost per DOAC claim in 2015 was $367.4 (interquartile range 323.9 to 445.9), as opposed to $12.3 (interquartile range 9.2 to 16.5) for warfarin. The median cost per standardized (30-day supply) prescription was $317.0 (interquartile range 303.8 to 324.3) and $8.0 (6.7 to 9.8) for DOACs and warfarin, respectively. DOAC adoption differs by specialty. Cardiologists, cardiac electrophysiologists and orthopedics had the highest predicted DOAC share per 100 claims (53.8, 72.9 and 71.5, respectively in 2015); nephrologists, family practitioners and geriatricians the lowest (22.3, 21.5 and 20.7, respectively in 2015). The p90/p10 ratio and the median absolute deviation from the median varied across HRRs and correlated positively with the prevalence of stroke and atrial fibrillation in the Medicare population.

**Conclusions:**

DOACs have been increasing their share year-over-year, but adoption varies across specialties. In prevalent areas for stroke and atrial fibrillation, prescription dispersion magnifies, and this may signify a rapid adoption by top providers.

## Introduction

Warfarin sodium, an inhibitor of vitamin K-dependent clotting factors, has been the cornerstone of oral anticoagulation. Warfarin is the most prescribed vitamin K-antagonist worldwide, but outside the U.S., phenprocoumon and acenocoumarol are also prescribed (Europe). Warfarin use has the drawbacks of frequent blood monitoring, food and drug interactions and risk of gastrointestinal and central nervous system bleeding, factors that make its use more difficult and less safe especially in domiciliary patients [1, 2]. Four direct-acting oral anticoagulants (DOAC)- the thrombin inhibitor dabigatran, and Factor Xa inhibitors rivaroxaban, apixaban, edoxaban [3–6]—are now approved for non-valvular atrial fibrillation and venous thromboembolism treatment (all four), secondary VTE prevention (rivaroxaban, apixaban, dabigatran), prophylaxis from venous thromboembolism after knee or hip replacement (dabigatran, rivaroxaban, apixaban) [7]. A fifth DOAC, betrixaban, received FDA approval in 2017 for extended thromboprophylaxis in acutely ill medical patients [8].

Warfarin remains the standard of care for patients with mechanical heart valves [9]. The favorable profile of these medications has led to a revision of the guidelines, has changed the prescription patterns, and rendered them popular among patients and providers [10]. On the other hand, the introduction of DOACs in the US with the greater quality-adjusted life expectancy may not represent a good “value for money” policy [11], as DOACs are associated with increased costs which influences prescription patterns and adherence [12]. Since the most significant differences between the two treatments approaches rely on the ease of administration on the one hand *vs*. the increase of drug cost on the other hand, DOACs provide an example to study adaptation of new treatments between practitioners. In this study, we describe DOAC prescribing patterns for Medicare Part D providers, using claim and payment data available through Centers for Medicare and Medicaid Services (CMS). We also assessed the impact of DOAC introduction on the drug payment costs and the variation on the prescribing practices, across providers, specialties and hospital referral regions (HRRs).

## Methods

### Data sources and search strategy

Medicare part D is a U.S. government program, aiming to subsidize expenditures of prescription drugs and prescription drugs insurance premiums for Medicare beneficiaries. Medicare beneficiaries signed up for benefits under part A (hospital insurance) and /or part B (medical insurance) are eligible for part D coverage. Part D benefits are administered by insurance companies through a stand-alone Prescription Drug Plan that covers drugs only, or through a part C health plan (covering both part A and part B at minimum, plus additional costs that include also prescription drugs, known as Medicare Advantage Prescription Drug plan).

We used Medicare part D publicly available data to describe variation in oral anticoagulation prescribing and calculate the associated drug costs. This is a retrospective, cross-sectional analysis of Medicare part D prescriber public use files (PUF) and the corresponding summary files for 2013 to 2015 calendar year. The Part D Prescriber PUF contains Prescription Drug Event records submitted by Medicare Advantage Prescription Drug and by stand-alone Prescription Drug Plans. The PUF assigns prescription drug event information to each provider, using the National Provider Identifier. The National Provider Identifier is a unique 10-digit number assigned to health care providers by the CMS National Plan and Provider Enumeration System. It identifies providers in a standardized way throughout the healthcare system. The 2015 up-to-date data for drug prescriptions and costs (released in 2017) were used for the main analysis whereas earlier 2013 and 2014 data served as comparison to explore changing prescription patterns. The CMS, is part of the Department of Health and Human Services dedicated to serve Medicare & Medicaid beneficiaries, aiming to strengthen health care services and information available to beneficiaries and health care providers. Among other activities, CMS offers a broad spectrum of quantitative data for researches and healthcare professionals, including claims data and drug spending. The data derive from the CMS Chronic Condition Data Warehouse, a database covering 100% of Medicare enrolment and Part D prescription drug data. All files are publicly available at the CMS website (Table A in S1 Supplementary appendix).

We used the online CMS tool to search for prescription claims for “dabigatran”, “rivaroxaban”, “apixaban”, “edoxaban”, and “warfarin”. We cross-validated results by repeating the extraction and analysis using the brand drug name “Pradaxa”, “Xarelto”, “Eliquis” and “Savaysa” for DOACs; “Coumadin”, “Jantoven” and “Warfarin Sodium” for warfarin claims (Table B in S1 Supplementary appendix). PDZ and ISK used the CMS search tool independently, including generic names and brand names as search terms, to constrain datasets to oral anticoagulants. We compared the extracted data files, the compiled tables and the analyses outputs, and were 100% match.

We used part D Drug National Summary table to pool claims and payments for oral anticoagulants over 2013 to 2015 calendar years and report their relative changes.

### Measures of prescription use and expenditure

We analyzed prescriber and summary PUFs to calculate anticoagulant claims, drug cost and prescribing dispersion across providers, specialties and regions. We collapsed data at the provider level using the National Provider Identifier and stratified results by medical specialty, by drug class (DOACs or warfarin) and claim year. We modeled DOAC claims as a count outcome via a truncated negative binomial regression. Poisson and negative binomial regression are used to model count outcomes, in settings where the dependent variable is a positive integer or zero. Truncated Poisson and truncated negative binomial regression are used to estimate model parameters by maximum likelihood, when the dependent variable (DOAC claims in our data) is a positive count whose values are all above a truncation point (that is 10 claims in our setting). Our data are truncated because there are no observations for providers with fewer than 11 claims. CMS records with 10 or fewer claims are suppressed to protect beneficiary privacy. This approach was preferred over summarizing the observed data, to allow correcting for left truncation of PUF claim data using maximum-likelihood estimation. Poisson regression assumes that the variance function equals the mean in the model. Within the framework of generalized linear models, the truncated Poisson model was run first and model fit testing was performed. As variance was larger than the mean, over-dispersion was present and the truncated negative binomial regression was preferred instead, because it extends Poisson properties to allow modelling the over-dispersion. We used the model estimates to generate the prescriber’s predicted profile (predicted counts) to calculate novel anticoagulant use by specialty and year. We used the predicted DOAC claims (provider-level counts) to describe variability in prescribing patterns [13].

### Regional variation of prescribing patterns

Higher and lower volume prescribers comprise metaphorically the two edges of a rubber band, whose edges can approach or pull apart, denoting decreasing or increasing dispersion [14]. We assigned PUF prescriber data to primary practice location and HRR, by the Dartmouth Atlas of Healthcare (Table A in S1 Supplementary appendix). We calculated 90/10 percentile (p90/p10) ratios as measures of prescribing dispersion for DOACs and the median absolute deviation from the median by HRRs [15]. These measures can provide a broad sense of the shape of disparity, and can provide robust non-parametric estimators of prescribing inequalities between prescribers; median absolute deviation from the median is a robust measure of spread resilient to outliers [16]. As such, these measures can highlight variation in DOAC prescribing by geographic region, over calendar years. We divided HRRs into quintiles based on the prevalence of target conditions (stroke and atrial fibrillation available at the CMS website, Table A in Supplementary appendix) among Medicare population, and p90/p10 percentile ratios of DOAC prescribing. We then tabulated quintiles of p90/p10 ratios by quintiles of target conditions, to evaluate the pattern of variation across HRRs, that is to associate prescribing dispersion for DOAC with the burden of target condition at the regional level. We used Stata (StataCorp LP, College Station, TX) for data analysis.

## Results

The descriptive data are illustrated in Table 1. In 2015, oral anticoagulant claims added up to approximately 24.4 million and incurred a payment cost of approximately $3.3 billion. These figures correspond to approximately 1.7% of all drug claims and 2.4% of all Medicare part D drug payments in 2015. DOACs comprised approximately 31.0% of all anticoagulant claims in 2015 PUF. That was mainly due to a 127% relative increase in 2015 DOAC claims, as opposed to 8.2% relative decline of warfarin claims, compared to 2013 calendar year. Overall, total payments for oral anticoagulants were approximately 2.5 times higher in 2015 ($3.3 vs. $1.3 billion for 2013), and comprised 2.4% of all part D drug payments (compared to 1.3% for 2013 claim year). DOAC claim payments tripled in 2015 (approximately $3.0 billion), compared to 2013 (approximately $1.1 billion), whereas warfarin 2015 payments showed a relative stability totaling $240.8 million, a mere 1.4% decline compared to 2013.

**Table 1:** Medicare part D oral anticoagulation usage and payments between 2013 and 2015 claim years.

**Total claims for oral anticoagulants (% of total part D claims)**		**%change**
2015	24,375,428 (1.7)	+12.7
2014	23,079,575 (1.6)	+6.7
2013	21,635,595 (1.6)	referent
Claims for DOACs (% of total claims for oral anticoagulants)		
2015	7,560,430 (31.0)	+ 127.3
2014	5,497,753 (23.8)	+ 65.3
2013	3,326,389 (15.4)	referent
Claims for warfarin (% of total claims for oral anticoagulants)		
2015	16,814,998 (69.0)	- 8.2
2014	17,581,822 (76.2)	- 4.0
2013	18,309,206 (84.6)	referent
**Total cost of oral anticoagulants in $ millions (% total part D drug payments)**		
2015	$ 3,276.8 (2.4)	+ 150.1
2014	$ 2,184,7 (1.8)	+ 66.8
2013	$ 1,310.0 (1.3)	referent
Cost for DOACs (% of total drug cost for oral anticoagulants)		
2015	$ 3,036.0 (92.7)	+ 184.8
2014	$ 1,964.5 (89.9)	+ 84.3
2013	$ 1,065.9 (81.4)	referent
Cost for warfarin (% total of total drug cost for oral anticoagulants)		
2015	$240.8 (7.3)	- 1.4
2014	$ 220,1 (10.1)	- 9.8
2013	$ 244.1 (18.6)	referent

Data pooled from part D Drug National Summary tables, 2013–2015 (Table A in S1 Supplementary appendix); DOACs = direct oral anticoagulants

We analyzed part D utilization and payment data restricting the PUF dataset to include provider claims for oral anticoagulant drugs. Part D utilization and payment data PUFs excludes records derived from providers with 10 or fewer claims to protect beneficiary privacy, resulting in underestimating the pooled claims by 9.0% and costs by 16.6% in 2015 (8.5% and 17.7%, respectively in 2014; 7.8% and 19%, respectively in 2013). Upper decile of providers prescribed approximately 9.9 million claims on oral anticoagulants in 2015, representing a net increase of 1.3 million claims since 2013. They filled approximately 3.2 million claims for DOAC, nearly 1.9 million higher relative to 2013 calendar year, while warfarin claims were approximately 0.6 million fewer. Consequently, top prescribers incurred total drug payments of approximately 1.4 billion (approximately half of all anticoagulant drug spending in 2015), adding an excess of approximately $ 845 million in drug payments compared to 2013 (S1 Fig).

Providers that prescribed for DOACs in 2015, incurred a median 30-day supply cost of $317.0 (interquartile range (IQR), $303.8 to $324.3) and a median payment per claim of $367.4 (IQR $323.9 to $445.9). Conversely, the median monthly prescription cost of warfarin was $8.0 (IQR $6.7 to $9.8), with a median cost per claim of $12.3 (IQR $9.2 to $16.5). That is, the prescription cost per standardized (30-day supply) prescription was nearly forty times higher for DOAC, compared to warfarin in 2015 (2013 and 2014 averages in Table C of S1 Supplementary appendix).

**Fig 1:**
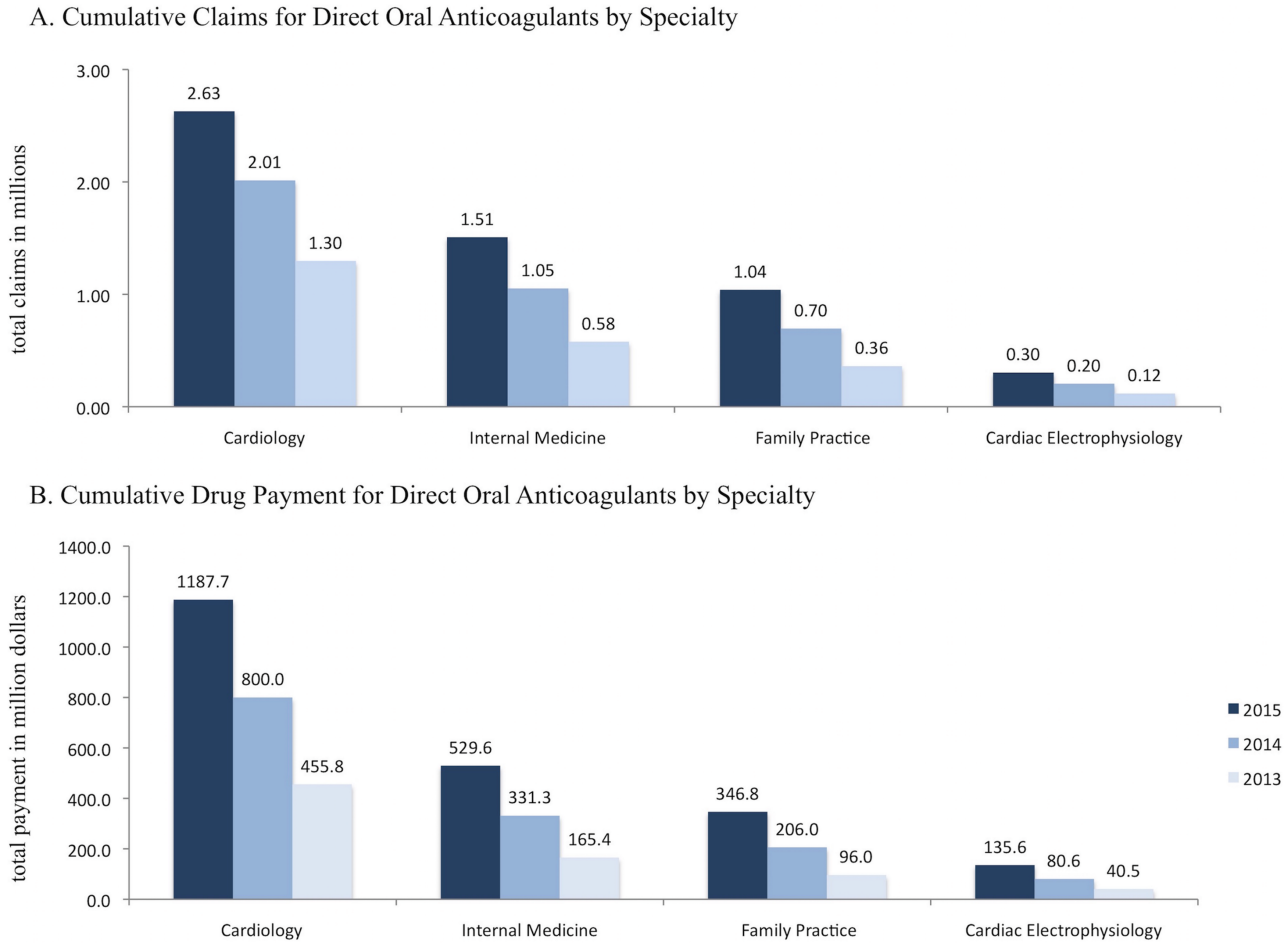
**Panel A shows the cumulative part D claims (in millions) for direct oral anticoagulants, stratified by calendar year. Panel B shows the cumulative part D payments (in million dollars) for direct oral anticoagulants, stratified by calendar year**. The top 4 specialties are displayed. Cumulative claims and costs in part D prescriber PUF underestimate claims and costs, as records with 10 or fewer claims are suppressed by CMS policy. Cardiology and interventional cardiology are merged to compare across calendar years (since the new specialty code for interventional cardiology to distinguish from clinical cardiology became officially effective in 2015).

The top 2015 prescribers of DOACs (ranked by cumulative claims) were cardiology (including interventional cardiology), internal medicine, family practice and cardiac electrophysiology. These four categories filled approximately 5.5 million (86%) of DOACs claims (Fig 1A), which accounted for approximately $2.2 billion (87% of pertinent drug payment cost) in prescriber PUF (Fig 1B). Noteworthy, internal medicine and family practice were the top prescribers regarding all anticoagulant claims (approximately 7.1 and 5.8 million claims, respectively in 2015), with a predominance of warfarin claims.

**Fig 2:**
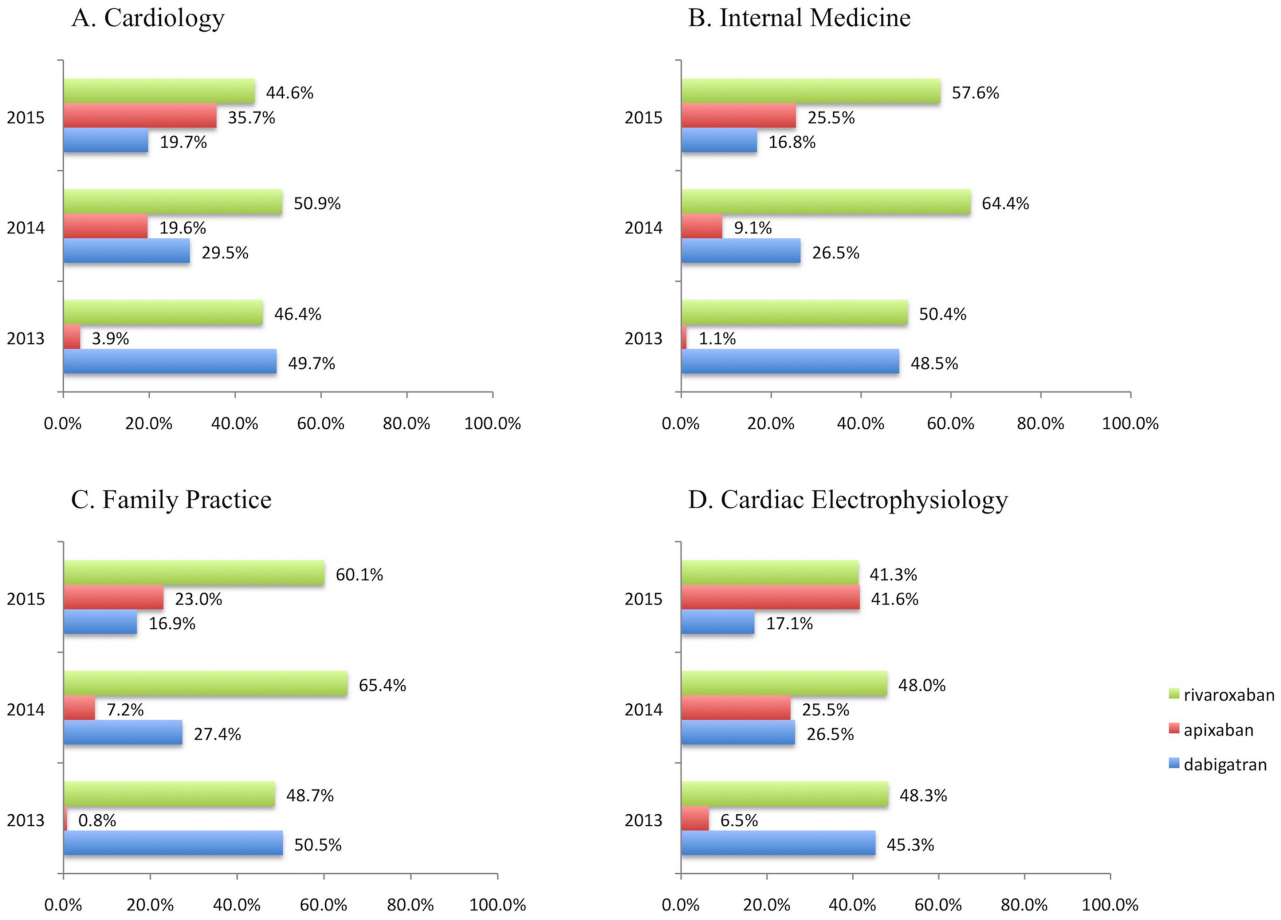
Share by drug per 100 standardized (30-day supply) prescriptions for direct oral anticoagulants (DOAC) in part D PUF, over 2013 to 2015 calendar years. The top 4 specialties by DOAC claims are displayed: (A) Cardiology, (B) Internal Medicine, (C) Family Practice, (D) Cardiac Electrophysiology. Cardiology and interventional cardiology are merged to compare across calendar years (since the new specialty code for interventional cardiology to distinguish from clinical cardiology became officially effective in 2015). Results are rounded to the first decimal. Edoxaban received FDA approval in 2015 and had no claims for 2013 and 2014 calendar years; it had fewer than <0.1% in 2015 part D PUF and does not appear in the figure.

Dabigatran and rivaroxaban dominated DOAC prescriptions in 2013, having approximately an equal percent share of standardized (30-day supply) prescriptions. Apixaban, which was approved in December 2012, had a low share in 2013, but increased in 2014 and 2015, while dabigatran share demonstrated a relative decline. Edoxaban was approved in 2015, and had only few claims, accounting for <0.1% of DOAC monthly prescriptions in 2015. Rivaroxaban reached a peak in 2014, but then showed a marginal decline in 2015 due to apixaban increase in share (Fig 2).

**Fig 3:**
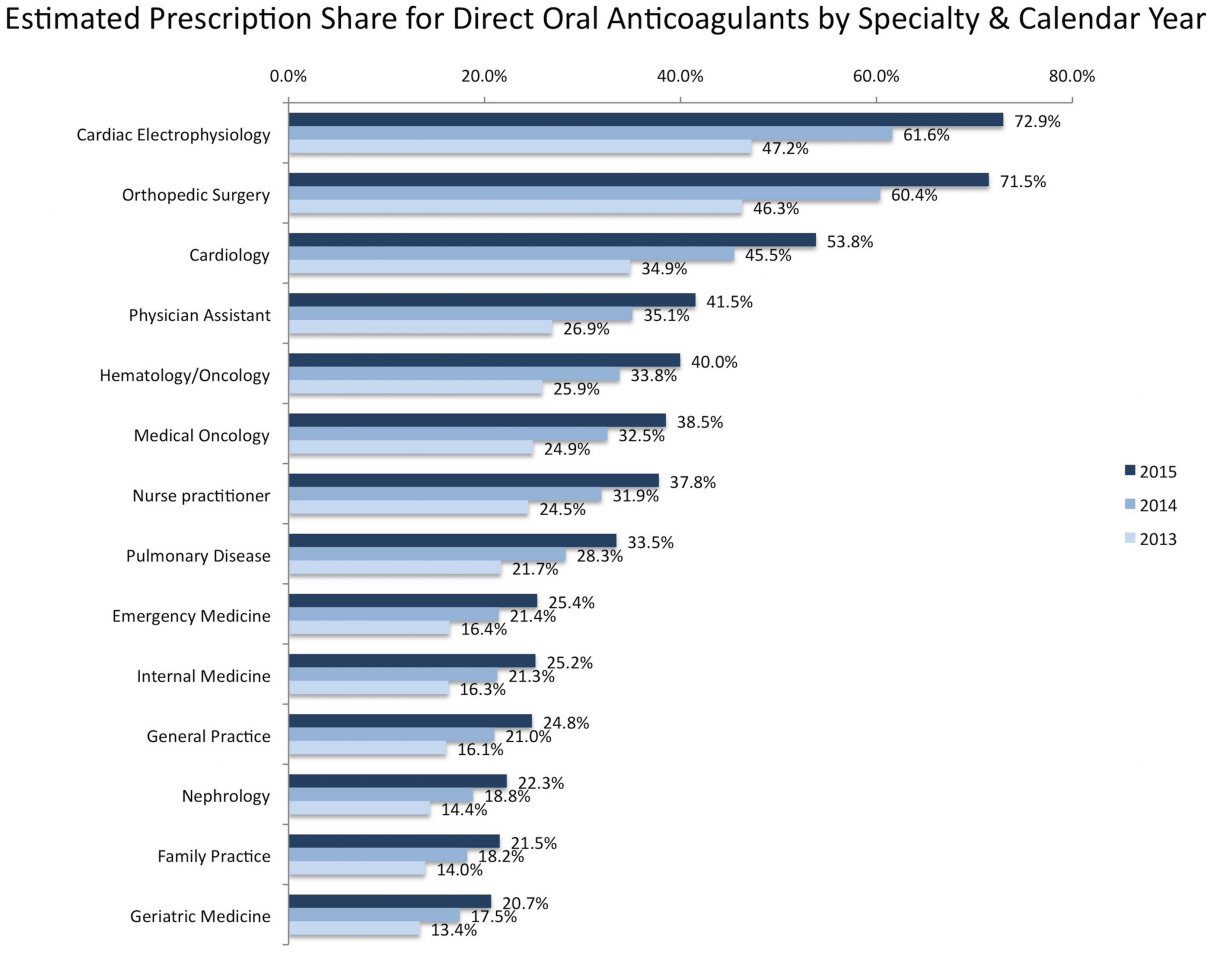
Estimated share for direct oral anticoagulants (DOAC), per 100 claims for oral anticoagulants in part D PUF. Predictions ranked in descending order by specialty classification, over 2013 to 2015 calendar years. The figure includes coded specialties with a minimum of 1,000 providers each (the 14 classes comprise approximately 97.4%, 97.5% and 97.4 of all part D claims for oral anticoagulants in 2013, 2014 and 2015 calendar years PUFs, respectively; Table D in S1 Supplementary appendix). Estimates derive from the predicted DOAC claim counts per provider, modeled via a truncated negative binomial regression, after adjusting for specialty classification and calendar year. The count of total anticoagulant claims per provider was set as exposure. The model accounts for left truncation (suppression of records with 10 or fewer claims by CMS policy) and generates predicted estimates via maximum likelihood estimation for the full dataset.

The predicted DOAC share, measured as DOAC claims per 100 total claims for oral anticoagulants, has increased consistently throughout the study period, suggesting the rapid adoption of DOACs. Specifically, across specialties, cardiac electrophysiology, orthopedic surgery and cardiology occupied top 3 places by DOAC share, while nephrology, family practice and geriatric medicine had the lowest predicted share (Fig 3).

**Fig 4:**
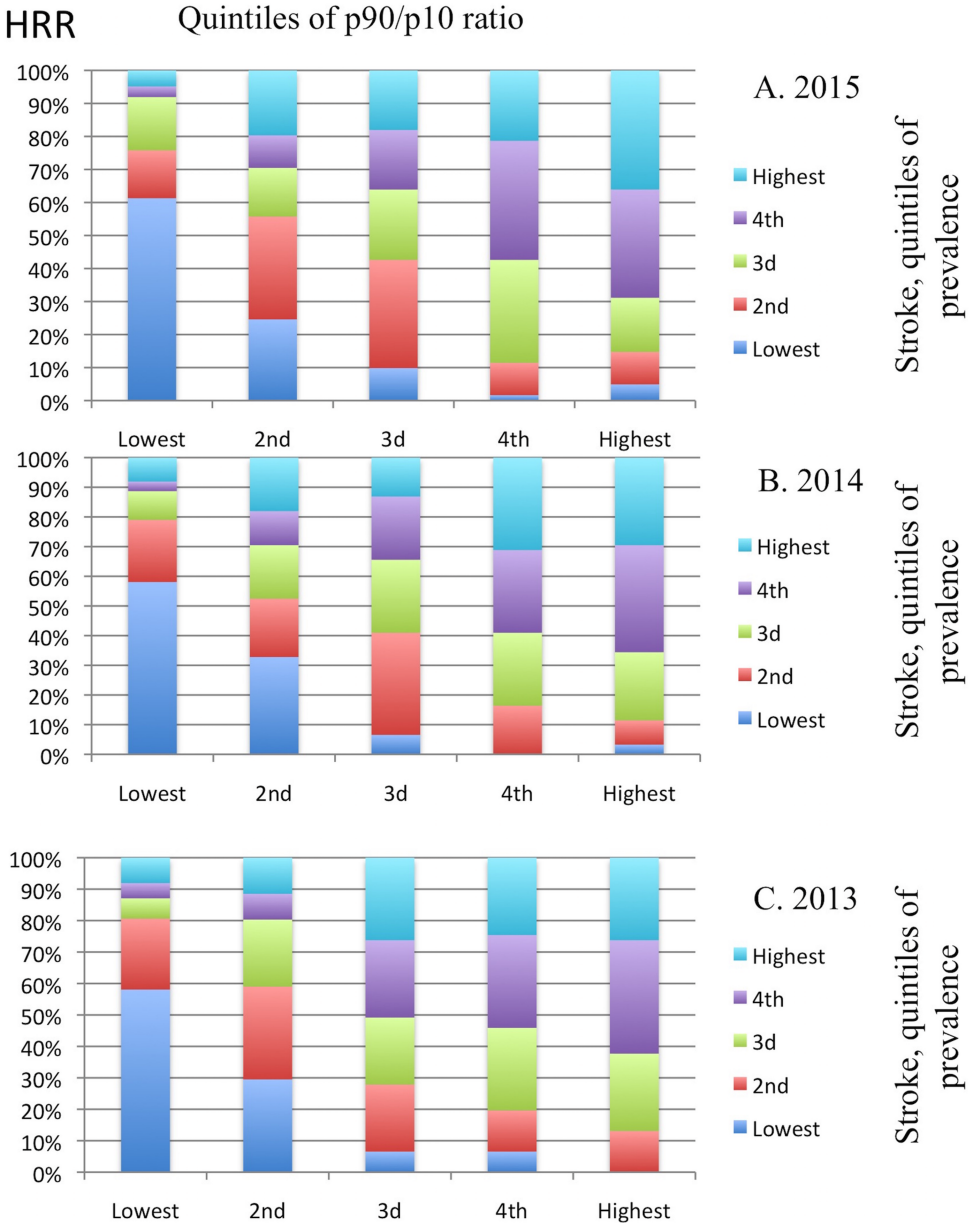
Quantile analysis of p90/p10 percentile ratios for direct oral anticoagulant (DOAC) claims *vs*. stroke prevalence among hospital referral regions (HRRs). Each column in the graph marks a separate quintile of p90/p10 ratio in consecutive order, from the lowest (1^st^) to the highest (5^th^). The quintiles of stroke prevalence are marked with discrete colored bars, from the lowest (indicated with blue bar) to the highest (indicated with light blue bar) and the size of each color bar represents the percent share within each quintile of HRRs p90/p10 ratio. The differences in DOACs claims between high volume and low volume prescribers (measured by the p90/p10 ratio) were more pronounced in regions where stroke was more prevalent. For example, 4.8% of HRRs in the lowest quintile for p90/p10 ratio belonged to the highest quintile of stroke (light blue bar) in 2015 (panel A); 36.1% of HRRs in the highest quintile for p90/p10 ratio belonged to the highest quintile of stroke (light blue bar). These figures were 8.1% and 29.5%, respectively in 2014 (panel B) and 8.1% and 26.2%, respectively for 2013 (Panel C). Regional variation exists in DOAC prescribing, being more dispersed in areas with higher prevalence of stroke, as these regions are aligned towards higher p90/p10 ratios (the full set of conditional probabilities are in Tables E to G in S1 Supplementary appendix).

We used p90/p10 percentile ratios of predicted DOAC claims across providers to highlight prominent differences across HRRs over calendar years. In 2015, the p90/p10 ratio varied across regions from 10.2 (in Bend, OR) to 54.2 (in Amarillo, TX). To explore the association between prescribing differences and stroke prevalence across HRRs, we used quantile analysis to stratify ordered data into equal-sized subgroups. We divided HRRs in five subgroups (quintiles) based on p90/p10 values and in five subgroups (quintiles) based on the prevalence of stroke across HRRs. Fig 4 graphically illustrates the quintile distribution of the p90/p10 percentile ratios *vs*. the quintile distribution for stroke prevalence among HRRs. That is, for HRRs in each quintile of p90/p10 ratio for DOAC claims, we show the share of HRRs from the lowest to the highest quintile for stroke prevalence. 4.8% of HRRs in the lowest quintile for p90/p10 ratio belonged to the highest quintile of stroke in 2015 (Fig 4A); 36.1% of HRRs in the highest quintile for p90/p10 ratio belonged to the highest quintile of stroke. These figures were 8.1% and 29.5%, respectively in 2014 (Fig 4B) and 8.1% and 26.2% in 2013 (Fig 4C). Conversely, 61.3% of HRRs in the lowest quintile for p90/p10 ratio belonged to the lowest quintile of stroke in 2015 (Fig 4A); 4.9% of HRRs in the highest quintile for p90/p10 ratio belonged to the lowest quintile of stroke. These figures were 58.1% and 3.2%, respectively in 2014 (Fig 4B) and 58.1% and 0% in 2013 (Fig 4C). That is, regional variation exists in DOAC prescribing, being more dispersed in areas with higher prevalence of the target conditions, as these regions are aligned towards higher p90/p10 ratios (numeric data available in Table E, Table F and Table G in S1 Supplementary appendix).

The HRR p90/p10 percentile ratios correlated strongly with stroke prevalence in 2015 (Spearman’s rho = +0.51, p < .001), and similar associations were found for 2014 (rho = +0.51, p < .001) and 2013 (rho = +0.50, p < .001). The HRR p90/p10 ratios correlated moderately with atrial fibrillation prevalence in 2015 (rho = +0.34, p < .001), and similar associations were found for 2014 (rho = +0.31, p < .001) and 2013 (rho = +0.31, p < .001).

The median absolute deviation from the median varied across HRRs from 5.1 (in Anchorage, AK) to 26.0 DOAC claims in 2015 (in Scranton, PA), indicating geographic variation in DOAC prescribing; the median absolute deviation from the median ranged from 4.0 to 20.8 claims in 2014, and 2.5 to 19.5 in 2013. The median absolute deviation from the median correlated moderately with atrial fibrillation (rho = +0.41, p < .001) but weakly with stroke distribution across HRRs (rho = +0.15, p = 0.01) in 2015. Similar associations were noted for previous calendar years (data not shown).

## Discussion

Time, practice and geography influence the adoption of DOACs. During the 2015 claim period, the use of DOAC mounted, totaling approximately 7.6 million claims and $3.0 billion for payments. Compared to 2013, part D prescriber PUF data, 2015 claims and cost for DOACs have multiplied by almost 3-fold. This expansion has led to an increased drug cost of approximately $2.0 billion for oral anticoagulation drugs; a dramatic effect that is due to the net increase in DOAC prescribing. The percentage change in DOACs prescriptions over the years is much larger than the change for warfarin, whose claims actually decline year-by-year. This shows that DOACs do not simply substitute warfarin, but rapidly increase their share as upfront treatment among previously untreated individuals. In fact it can be assumed, that DOACs are prescribed to oral anticoagulation-naïve patients that have a new diagnosis (e.g. atrial fibrillation) and/or they are prescribed to patients with indication for anticoagulation therapy who were not treated yet with an oral anticoagulant. However, this expansion occurs unevenly across medical disciplines, going down from more to less specialized, regarding the core indications for DOAC. More specifically in terms of claim volume, adoption is affected by type of practice: general practitioners, internists and family doctors will lag behind physicians that are more closely related to atrial fibrillation and VTE management, namely cardiologists and subspecialties, orthopedics and oncologists. Moreover, in high prevalent regions for stroke or atrial fibrillation, prescribing dispersion between top and low prescribers magnifies, indicating that top prescribers may have a prolific effect in DOAC adoption.

As a new class, the DOACs faced challenges in market diffusion. In general, the adoption of any new class of medications can be slow because of lack of physicians’ awareness, physicians’ expertise or comfort level or an intentionally initial cautious approach [17, 18]. Part D prescribing for DOACs is unevenly distributed among the medical specialties. Cardiology and subspecialties have led the DOAC expansion. In a previous study, cardiologists have been described as “rapid adopters” of the new medications regardless of the practice setting [17]. This is not surprising since atrial fibrillation, a major approved indication for long-term, oral anticoagulation is the leading cause of arrhythmia in the US, associated with increasing morbidity [19]. Atrial fibrillation incidence increases with age, hence the use of anticoagulants is high among Medicare beneficiaries [20]. A rapid adoption of DOAC prescribing has been described for the patients with atrial fibrillation with more patients treated with anticoagulant therapy since their introduction [21]. This rapid adoption has come with significant cost implications [22]. As a result of the projected rise in the number of the affected individuals to more than 5.6 million in 2050 (from 2.3 million in 2001), the anticoagulation cost will relentlessly increase [23]. Cardiologists and the subspecialties are more likely to see patients with atrial fibrillation who have a difficulty to maintain adequate anticoagulation control or they are in poor health statuses and therefore they are candidates for the newer treatments [17]. This can further explain the fact that these specialists lead the DOAC expansion.

Internists and family practitioners may refill the DOAC prescriptions that are initiated by cardiologists. It seems though that they are “slower adopters” and in a Canadian study the family medicine trainees may even prefer warfarin to DOACs despite the guidelines and recommendations [24]. Nephrologists, not unexpectedly, have a low share for DOAC, since their usage is limited by the potential of accumulation in patients with declining renal function [25].

The top 10% of the prescribers accounted for half of prescriptions for oral anticoagulants. This skewing also held true for the total costs contrasting with what has been reported for other classes of medications before [26]. Noteworthy, prescribing dispersion showed geographic variability across HRRs, being more prominent in areas with high prevalence of atrial fibrillation and stroke. The DOAC prescribing pattern resembles the “rubber effect” with one end representing the low volume prescribers and the other end the high-volume ones. High volume prescribers have a greater opportunity to prescribe new drugs [18], “stretching” the gap. This is expected since they are probably exposed to a greater volume of eligible population and they may also be targeted by the pharmaceutical industry in a higher degree. The spread of DOAC claims across regions correlates with atrial fibrillation burden and stroke prevalence, highlighting that prescribing dispersion across HRRs have a complex, multifactorial background. One factor is the different composition of health care providers within a region. That pertains to the individual physician’s medical practice and networking, but also specialty has a role in DOAC adoption. Additional factors are the burden of the target condition, which seems to drive prescription dispersion. Health and socioeconomic status of the target population also have a role, but were not analyzed in the current study which does not include patient-level data.

Another interesting finding is the increase in the apixaban and rivaroxaban use, which was accompanied with a relative decrease in dabigatran prescribing. Apixaban was introduced later but it showed a pattern of rapid adoption. In a previous study on a different database, the IMS Health National Disease and Therapeutic Index, rivaroxaban was the top DOAC prescribed in outpatient office visits up to 2014 [21]. This holds true for part D standardized monthly prescriptions across top DOAC prescribers, as rivaroxaban reached a peak in 2014, but then showed a marginal decline in 2015 as apixaban increased its share.

The relative share of each DOAC, follows the dissemination of evidence across medical specialties and is influenced by marketing policies and promotion by the industry. However, in real-world clinical practice minor differences in cost, efficacy and safety and/or ease of administration may exert a significant impact in drug selection. In terms of cost for example, dabigatran may have the lower all-cause costs (including monthly-per-patient cost and healthcare utilization) compared to apixaban, rivaroxaban and warfarin [27]. Notably, the agents with the higher renal elimination (dabigatran and edoxaban), may be less preferred compared to rivaroxaban and apixaban. Apixaban and rivaroxaban have an established dose modification scheme for patients with moderate chronic renal failure. In turn, apixaban may preferred over rivaroxaban, as rivaroxaban should be taken with a large meal, whereas there is no such restriction for apixaban [7]. Apixaban may outperform competitors, when efficacy, safety and cost are jointly evaluated [28]. Drug selection will also depend on the particular clinical endpoint set by the physician: for example a cardiologist may consider bleeding as the major outcome to avoid when treating a patient with atrial fibrillation who needs a percutaneous coronary intervention. In that setting, rivaroxaban has an established safety profile from randomized data [29].

The “DOAC era” may have come at an increased cost for patients and providers, however there is supporting evidence that their safety and efficacy profile combined with the lack of need of for monitoring may be cost-effective compared to warfarin [28]. In a recent U.S. study of newly treated, non-valvular atrial fibrillation patients, warfarin lagged behind DOACs in terms of total healthcare cost, including inpatient and outpatient costs [27]. A recent evidence-synthesis and cost-effectiveness analysis conducted in the U.K., reported that DOACs outperform warfarin in major outcomes, including the risk of stroke, the risk of systemic embolism, all-cause mortality, major bleeding and intracranial bleeding. Furthermore, in a lifetime horizon simulation of a typical patient aged 70 years with atrial fibrillation who starts oral anticoagulants, warfarin was unlikely to be cost-effective [28].

Our study is limited by lack of generalizability as it has also been described in previous studies [30]. Commercial claims databases [30] may not reflect practices across Medicare beneficiaries and vice-versa [20]. Data on outpatient visits may lack patient-level factors and exclude emergency department care and outpatient clinics [21]. The data we analyzed reflect the prescribing patterns of a comprehensive national population of Medicare Part D prescribers but they lack any insight in providers’ complete practices and in patient-level data. As a result, they cannot be used to address the quality of prescribing patterns or behaviors and to draw conclusions on the quality of care for both prescribers and individual patients, since there are no patient-level data in this study. The PUF includes providers treating part D beneficiaries and may not disclose how providers treat non-Medicare patients and commercially insured patients. Moreover, the data may not reflect a provider’s entire practice or all of Medicare, as it only includes information on beneficiaries enrolled in the Medicare Part D prescription drug program, approximately two-thirds of all Medicare beneficiaries [31]. Ranking for drugs and cost in total and by specialty may not apply for non-Medicare population, because certain conditions such as atrial fibrillation preferentially affect older populations. Also, our study did not examine how the patient’ out-of-pocket expenses (which can vary based on depend on which benefits phase each patient is) affected the type of anticoagulant initiated. However, in a previous study patients who had a supplementary coverage were may be more likely to be prescribed DOACs [20].

Our study reveals the exploding expansion of DOACs in the U.S., which should be viewed within the broader scope of the ever-increasing cost of medical care. Strategy changes such as careful patient selection, generic substitutions (when possible) as first-line agents and direct price negotiation (when allowed) should be encouraged.

## Supporting information

S1 Supplementary appendix The appendix includes the following items: Table A detailed list of public use files (PUFs) used for data analysis; Table B, the search terms for part D prescriber drugs public analytic files (PUFs), used to extract oral anticoagulation claims; Table C, the Medicare part D average prescription cost per provider for oral anticoagulants; Table D, the prescribers of oral anticoagulants in part D PUF, by specialty description; Table E, the dispersion between higher and lower percentile prescribers (for DOAC claims) *vs*. stroke prevalence among hospital referral regions in 2015: Quantile analysis; Table F, the dispersion between higher and lower percentile prescribers (for DOAC claims) *vs*. stroke prevalence among hospital referral regions in 2014: Quantile analysis; Table G, the dispersion between higher and lower percentile prescribers (for DOAC claims) *vs*. stroke prevalence among hospital referral regions in 2013: Quantile analysis.
(DOCX)

S1 Fig Top 10% of part D prescribers for oral anticoagulants, 2013–2015 calendar years. Panel A shows the cumulative claims (in millions; y-axis) for oral anticoagulants, stratified by class, namely Direct Oral Anticoagulants (DOAC) or warfarin (W), over 2013 to 2015 calendar years. Panel B shows the cumulative payment (in million dollars; y-axis) for oral anticoagulants over 2013 to 2015 calendar years. Top part D prescribers 1% to 10% on x-axis. Part D utilization and payment data PUFs excludes records derived from providers with 10 or fewer claims to protect beneficiary privacy, resulting in underestimating the pooled costs and claims.
(TIFF)
