# Supplementary material for: Medicare part D prescribing for direct oral anticoagulants in the United States: Cost, use and the “rubber effect”
Source: PLoS One. 2018 Jun 7;13(6):e0198674. doi: 10.1371/journal.pone.0198674 (PMC5991647; doi:10.1371/journal.pone.0198674)
Supplement: S1 Supplementary appendix — The appendix includes the following items: Table A detailed list of public use files (PUFs) used for data analysis; Table B, the search terms for part D prescriber drugs public analytic files (PUFs), used to extract oral anticoagulation claims; Table C, the Medicare part D average prescription cost per provider for oral anticoagulants; Table D, the prescribers of oral anticoagulants in part D PUF, by specialty description; Table E, the dispersion between higher and lower percentile prescribers (for DOAC claims) vs. stroke prevalence among hospital referral regions in 2015: Quantile analysis; Table F, the dispersion between higher and lower percentile prescribers (for DOAC claims) vs. stroke prevalence among hospital referral regions in 2014: Quantile analysis; Table G, the dispersion between higher and lower percentile prescribers (for DOAC claims) vs. stroke prevalence among hospital referral regions in 2013: Quantile analysis. (DOCX) [file pone.0198674.s001.docx]

**SUPPLEMENTARY APPENDIX**

This appendix has been provided by the authors to give readers additional information about their work. Supplement to: Ziakas PD, Kourbeti IS, Poulou LS, Vlachogeorgos GS, Mylonakis E. Medicare Part D prescribing for direct oral anticoagulants in the United States: Cost, use and the “rubber effect”

| **Table of Contents** |
| --- |
| Detailed list of public use files (PUFs) used for data analysis (Table A) |
| Search terms for part D prescriber drugs public analytic files (PUFs), used to extract oral anticoagulation claims (Table B) |
| Medicare part D average prescription cost per provider for oral anticoagulants (Table C) |
| Prescribers of oral anticoagulants in part D PUF, by specialty description (Table D) |
| Dispersion between higher and lower percentile prescribers (for DOAC claims) *vs.* stroke prevalence among hospital referral regions, 2015: Quantile analysis (Table E) |
| Dispersion between higher and lower percentile prescribers (for DOAC claims) *vs.* stroke prevalence among hospital referral regions, 2014: Quantile analysis (Table F) |
| Dispersion between higher and lower percentile prescribers (for DOAC claims) *vs.* stroke prevalence among hospital referral regions, 2013: Quantile analysis (Table G) |

**Table A: Public Use Files (PUFs) used for data analysis**

| **#1 Description** | **Medicare Provider Utilization and Payment Data: 2015 Part D Prescriber & Drug National Summary Table** |
| --- | --- |
| Filenames | Medicare_Provider_Utilization_and_Payment_Data__2015_Part_D_Prescriber.csv  PartD_Prescriber_PUF_Drug_Ntl_15.xlsx |
| Available at: | The Centers for Medicare & Medicaid Services (CMS)  <https://www.cms.gov/Research-Statistics-Data-and-Systems/Statistics-Trends-and-Reports/Medicare-Provider-Charge-Data/PartD2015.html> |
| Date created | May 25, 2017 |
| **#2 Description** | **Medicare Provider Utilization and Payment Data: 2014 Part D Prescriber & Drug National Summary Table** |
| Filenames | Medicare_Provider_Utilization_and_Payment_Data__2014_Part_D_Prescriber.csv  PartD_Prescriber_PUF_Drug_Ntl_14.xlsx |
| Available at: | The Centers for Medicare & Medicaid Services (CMS)  <https://www.cms.gov/Research-Statistics-Data-and-Systems/Statistics-Trends-and-Reports/Medicare-Provider-Charge-Data/PartD2014.html> |
| Date created | May 13, 2017 |
| **#3 Description** | **Medicare Provider Utilization and Payment Data: 2013 Part D Prescriber & Drug National Summary Table** |
| Filenames | Medicare_Provider_Utilization_and_Payment_Data__2013_Part_D_Prescriber.csv  PartD_Prescriber_PUF_Drug_Ntl_13.xlsx |
| Available at: | The Centers for Medicare & Medicaid Services (CMS)  <https://www.cms.gov/Research-Statistics-Data-and-Systems/Statistics-Trends-and-Reports/Medicare-Provider-Charge-Data/PartD2013.html> |
| Date created | May 12, 2017 |
| **#4 Description** | **Medicare Provider Utilization and Payment Data: Medicare Part D Grand Totals and Overall Averages CY2015** |
| Filename | PartD_Prescriber_PUF_Grand_Totals_15.xlsx |
| Available at: | The Centers for Medicare & Medicaid Services (CMS)  <https://www.cms.gov/Research-Statistics-Data-and-Systems/Statistics-Trends-and-Reports/Medicare-Provider-Charge-Data/PartD2015.html> |
| Date created | May 24, 2017 |
| **#5 Description** | **Medicare Provider Utilization and Payment Data: Medicare Part D Grand Totals and Overall Averages CY2014** |
| Filename | PartD_Prescriber_PUF_Grand_Totals_14.xlsx |
| Available at: | The Centers for Medicare & Medicaid Services (CMS)  <https://www.cms.gov/Research-Statistics-Data-and-Systems/Statistics-Trends-and-Reports/Medicare-Provider-Charge-Data/PartD2014.html> |
| Date created | May 24, 2017 |
| **#6 Description** | **Medicare Provider Utilization and Payment Data: Medicare Part D Grand Totals and Overall Averages CY2013** |
| Filename | PartD_Prescriber_PUF_Grand_Totals_13.xlsx |
| Available at: | The Centers for Medicare & Medicaid Services (CMS)  <https://www.cms.gov/Research-Statistics-Data-and-Systems/Statistics-Trends-and-Reports/Medicare-Provider-Charge-Data/PartD2013.html> |
| Date created | May 24, 2017 |
| **#7 Description** | **Chronic Conditions** |
| Filenames | HRR_Table_Chronic_Conditions_Prevalence_by_Age_2015.xlsx  HRR_Table_Chronic_Conditions_Prevalence_by_Age_2014.xlsx  HRR_Table_Chronic_Conditions_Prevalence_by_Age_2013.xlsx |
| Available at: | The Centers for Medicare & Medicaid Services (CMS)  <https://www.cms.gov/Research-Statistics-Data-and-Systems/Statistics-Trends-and-Reports/Chronic-Conditions/CC_Main.html> |
| Date created | Dec 21, 2016 |
| **#8 Description** | **Geographic Boundary Files, Hospital Referral Regions (HRR) Boundary Files** |
| Filename | hrr_bdry.zip |
| Available at: | The Dartmouth Atlas of Healthcare  <http://www.dartmouthatlas.org/tools/downloads.aspx?tab=39> |
| Date created | January 13, 2005 |
| **#9 Description** | **Geographic Crosswalks and Research Files, ZIP code Crosswalks to Hospital Referral Regions (HRR)** |
| Filenames | ZipHsaHrr15.xls; ZipHsaHrr14.xls; ZipHsaHrr13.xls; |
| Available at: | The Dartmouth Atlas of Healthcare  <http://www.dartmouthatlas.org/tools/downloads.aspx?tab=39> |

**Table B: Search terms used to limit part D Prescriber Data PUF to anticoagulation claims**

|  | analysis using drug generic name | validation using drug brand name |
| --- | --- | --- |
|  | CMS coding variable | |
|  | **generic_name** | **drug_name** |
| Term #1 | DABIGATRAN ETEXILATE MESYLATE | PRADAXA |
| Term #2 | APIXABAN | ELIQUIS |
| Term #3 | RIVAROXABAN | XARELTO |
| Term #4 | EDOXABAN TOSYLATE | SAVAYSA |
| Term #5 | WARFARIN SODIUM | JANTOVEN |
| Term #6 |  | COUMADIN |
| Term #7 |  | WARFARIN SODIUM |

**Table C: Medicare part D average prescription cost for oral anticoagulants**

|  | **Median payment per 30-day supply, $**  **(interquartile range)** | **Median payment per claim, $ (interquartile range)** |
| --- | --- | --- |
| **2015** |  |  |
| DOAC | 317.0 (303.8 to 324.3) | 367.4 (323.9 to 445.9) |
| Warfarin | 8.0 (6.7 to 9.8) | 12.3 (9.2 to 16.5) |
| **2014** |  |  |
| DOAC | 285.0 (272.9 to 292.6) | 325.1 (291.7 to 393.6) |
| warfarin | 7.0 (5.9 to 8.6) | 10.5 (8.0 to 13.9) |
| **2013** |  |  |
| DOAC | 257.1 (246.6 to 263.9) | 289.9 (263.4 to 351.9) |
| warfarin | 7.9 (6.6 to 9.7) | 11.4 (8.8 to 15.0) |

DOAC = direct oral anticoagulants

**Table D: Prescribers of oral anticoagulants in part D public use files (PUF), by specialty description**

|  |  | **2013** | **2014** | **2015** |
| --- | --- | --- | --- | --- |
| **rank#** | **Specialty Description** |  |  |  |
| **1** | Family Practice | 58,172 | 58,267 | 58,265 |
| **2** | Internal Medicine | 57,194 | 57,056 | 56,741 |
| **3** | Nurse Practitioner | 18,412 | 20,726 | 23,505 |
| **4** | Cardiology | 18,965 | 19,350 | 19,546 |
| **5** | Physician Assistant | 10,123 | 10,856 | 11,573 |
| **6** | Hematology/Oncology | 3,853 | 4,067 | 4,225 |
| **7** | General Practice | 3,636 | 3,489 | 3,377 |
| **8** | Orthopedic Surgery | 3,075 | 2,677 | 2,207 |
| **9** | Nephrology | 2,492 | 2,293 | 2,124 |
| **10** | Pulmonary Disease | 1,718 | 1,738 | 1,748 |
| **11** | Cardiac Electrophysiology | 1,140 | 1,348 | 1,524 |
| **12** | Geriatric Medicine | 1,422 | 1,395 | 1,476 |
| **13** | Emergency Medicine | 1,429 | 1,306 | 1,170 |
| **14** | Medical Oncology | 1,043 | 1,076 | 1,152 |
|  | **Total No. of Providers** | **182,674** | **185,644** | **188,633** |
|  |  |  |  |  |
|  | **Total No. of Providers** | **192,056** | **194,929** | **197,980** |
|  | **% coverage** | **95.1** | **95.2** | **95.3** |

Note: Specialty description ranking by number of providers in part D PUF. All specialties were used to summarize oral anticoagulant claims and costs. Publicly available files (PUFs) suppress records with <11 claims by CMS policy to protect patient privacy; pooled providers, claims and cost in PUF underestimate actual claims and cost under part D.

Coded specialties with >1,000 providers each (14 classes, covering >95% of anticoagulant prescribers in PUF) were used to model direct oral anticoagulants (DOAC) predicted share across specialties.

**Table E: Dispersion between higher and lower percentile prescribers (for DOAC claims) across hospital referral regions, 2015: Quantile analysis**

**+-------------------+**

| Key | p90/10: Lowest quintile [10.19,16.68] ; 2^nd^ quintile [16.69,

|-------------------| 19.68] ; 3d quintile [19.71, 22.45] ; 4^th^ quintile

| frequency | [22.48 ,27.74]; Highest quintile [27.77 , 54.20]

| row percentage | stroke :Lowest quintile [2.24 ,3.14] ; 2^nd^ quintile [3.16 ,

| column percentage | 3.69] ; 3d quintile [3.70 , 4.05]; 4^th^ quintile [4.06

+-------------------+ 4.48] ; highest quintile [4.50 , 6.12]

**Hospital Referral Regions**

**quintiles | quintiles of p90/p10 ratio**

**of stroke** | 1 2 3 4 5 | Total

-----------+-------------------------------------------------------+----------

1 | 38 15 6 1 3 | 63

| 60.32 23.81 9.52 1.59 4.76 |

| 61.29 24.59 9.84 1.64 4.92 | 20.59

-----------+-------------------------------------------------------+----------

2 | 9 19 20 6 6 | 60

| 15.00 31.67 33.33 10.00 10.00 |

| 14.52 31.15 32.79 9.84 9.84 | 19.61

-----------+-------------------------------------------------------+----------

3 | 10 9 13 19 10 | 61

| 16.39 14.75 21.31 31.15 16.39 |

| 16.13 14.75 21.31 31.15 16.39 | 19.93

-----------+-------------------------------------------------------+----------

4 | 2 6 11 22 20 | 61

| 3.28 9.84 18.03 36.07 32.79 |

| 3.23 9.84 18.03 36.07 32.79 | 19.93

-----------+-------------------------------------------------------+----------

5 | 3 12 11 13 22 | 61

| 4.92 19.67 18.03 21.31 36.07 |

| 4.84 19.67 18.03 21.31 36.07 | 19.93

-----------+-------------------------------------------------------+----------

Total | 62 61 61 61 61 | 306

| 20.26 19.93 19.93 19.93 19.93 |

**Table F: Dispersion between higher and lower percentile prescribers (for DOAC claims) across hospital referral regions, 2014: Quantile analysis**

**+-------------------+**

| Key | p90/10: Lowest quintile [9.24,15.89] ; 2^nd^ quintile [15.95,

|-------------------| 18.73] ; 3d quintile [18.77, 21.43] ; 4^th^ quintile

| frequency | [21.46 ,25.82]; Highest quintile [25.88 , 55.66]

| row percentage | stroke :Lowest quintile [1.95 ,2.89] ; 2^nd^ quintile [2.93 ,

| column percentage | 3.43] ; 3d quintile [3.45 , 3.83]; 4^th^ quintile [3.84

+-------------------+ 4.27] ; highest quintile [4.29 , 5.28]

**Hospital Referral Regions**

**quintiles | quintiles of p90/p10 ratio**

**of stroke** | 1 2 3 4 5 | Total

-----------+-------------------------------------------------------+----------

1 | 36 20 4 0 2 | 62

| 58.06 32.26 6.45 0.00 3.23 |

| 58.06 32.79 6.56 0.00 3.28 | 20.26

-----------+-------------------------------------------------------+----------

2 | 13 12 21 10 5 | 61

| 21.31 19.67 34.43 16.39 8.20 |

| 20.97 19.67 34.43 16.39 8.20 | 19.93

-----------+-------------------------------------------------------+----------

3 | 6 11 15 15 14 | 61

| 9.84 18.03 24.59 24.59 22.95 |

| 9.68 18.03 24.59 24.59 22.95 | 19.93

-----------+-------------------------------------------------------+----------

4 | 2 7 13 17 22 | 61

| 3.28 11.48 21.31 27.87 36.07 |

| 3.23 11.48 21.31 27.87 36.07 | 19.93

-----------+-------------------------------------------------------+----------

5 | 5 11 8 19 18 | 61

| 8.20 18.03 13.11 31.15 29.51 |

| 8.06 18.03 13.11 31.15 29.51 | 19.93

-----------+-------------------------------------------------------+----------

Total | 62 61 61 61 61 | 306

| 20.26 19.93 19.93 19.93 19.93 |

**Table G: Dispersion between higher and lower percentile prescribers (for DOAC claims) across hospital referral regions, 2013: Quantile analysis**

+-------------------+

**+-------------------+**

| Key | p90/10: Lowest quintile [8.61,14.21] ; 2^nd^ quintile [14.38,

|-------------------| 17.59] ; 3d quintile [17.62, 20.07] ; 4^th^ quintile

| frequency | [20.10 ,24.10]; Highest quintile [24.11 , 50.97]

| row percentage | stroke :Lowest quintile [1.87 ,2.93] ; 2^nd^ quintile [2.95 ,

| column percentage | 3.46] ; 3d quintile [3.47 , 3.82]; 4^th^ quintile[3.84,

+-------------------+ 4.33] ; highest quintile [4.34 , 5.56]

**Hospital Referral Regions**

**quintiles | quintiles of p90/p10 ratio**

**of stroke** | 1 2 3 4 5 | Total

-----------+-------------------------------------------------------+----------

1 | 36 18 4 4 0 | 62

| 58.06 29.03 6.45 6.45 0.00 |

| 58.06 29.51 6.56 6.56 0.00 | 20.26

-----------+-------------------------------------------------------+----------

2 | 14 18 13 8 8 | 61

| 22.95 29.51 21.31 13.11 13.11 |

| 22.58 29.51 21.31 13.11 13.11 | 19.93

-----------+-------------------------------------------------------+----------

3 | 4 13 13 16 15 | 61

| 6.56 21.31 21.31 26.23 24.59 |

| 6.45 21.31 21.31 26.23 24.59 | 19.93

-----------+-------------------------------------------------------+----------

4 | 3 5 15 18 22 | 63

| 4.76 7.94 23.81 28.57 34.92 |

| 4.84 8.20 24.59 29.51 36.07 | 20.59

-----------+-------------------------------------------------------+----------

5 | 5 7 16 15 16 | 59

| 8.47 11.86 27.12 25.42 27.12 |

| 8.06 11.48 26.23 24.59 26.23 | 19.28

-----------+-------------------------------------------------------+----------

Total | 62 61 61 61 61 | 306

| 20.26 19.93 19.93 19.93 19.93 |

Note: We used the predicted DOAC claims (prescriber-level counts) to calculate 90/10 percentile ratios (p90/p10 ratios) as a measure of dispersion across providers, stratified by hospital referral regions (HRRs) where they practice. For each quintile of p90/p10 ratio, we show the quintile distribution of stroke prevalence among HRRs, for 2015 (Table E), 2014 (Table F) and 2013 (Table G). Data presented in tabular (numeric) form.
