# Supplementary figures and images for: Medicare part D prescribing for direct oral anticoagulants in the United States: Cost, use and the “rubber effect”
Source: PLoS One. 2018 Jun 7;13(6):e0198674. doi: 10.1371/journal.pone.0198674 (PMC5991647; doi:10.1371/journal.pone.0198674)

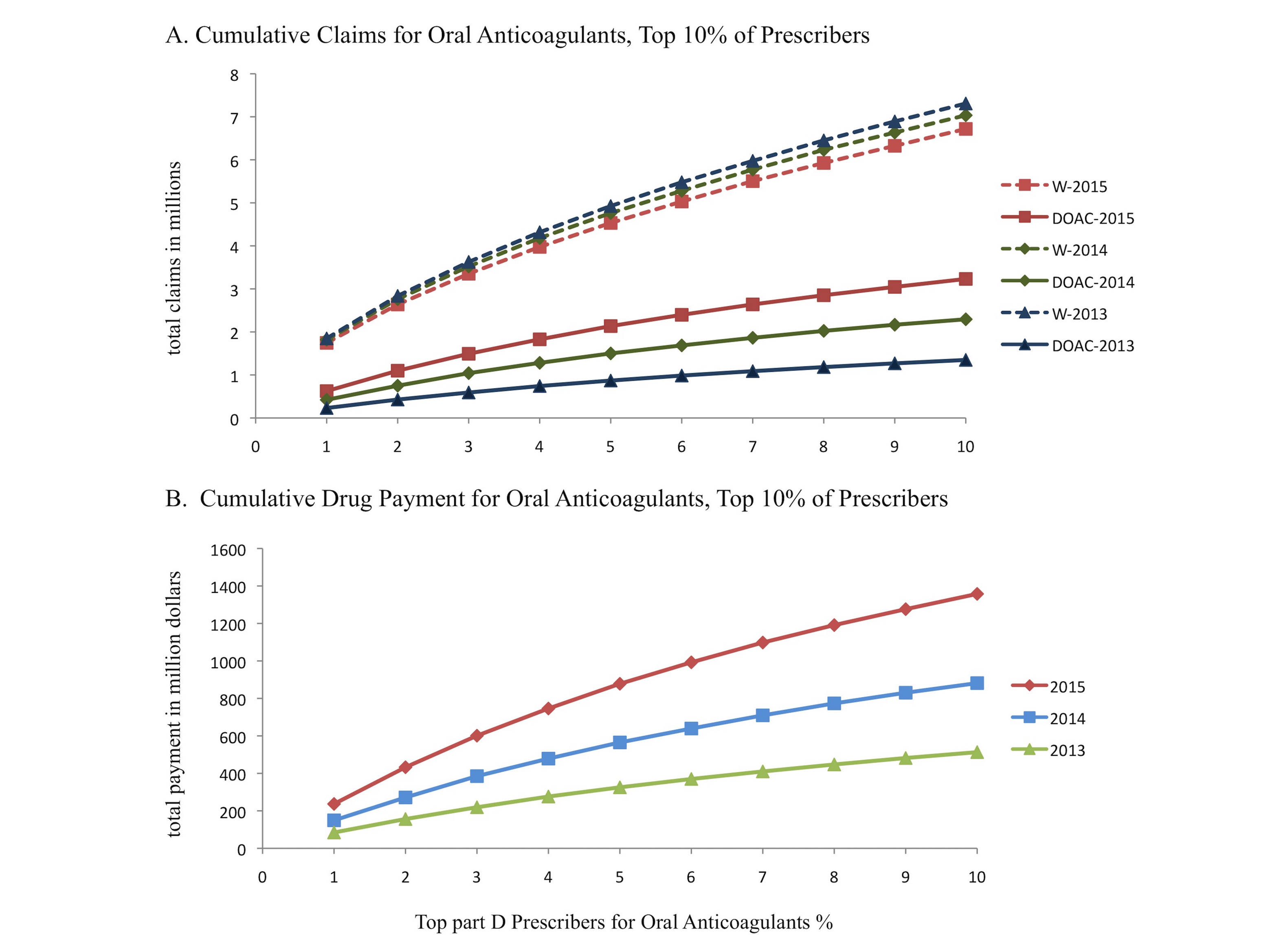

Supplement: S1 Fig — Panel A shows the cumulative claims (in millions; y-axis) for oral anticoagulants, stratified by class, namely Direct Oral Anticoagulants (DOAC) or warfarin (W), over 2013 to 2015 calendar years. Panel B shows the cumulative payment (in million dollars; y-axis) for oral anticoagulants over 2013 to 2015 calendar years. Top part D prescribers 1% to 10% on x-axis. Part D utilization and payment data PUFs excludes records derived from providers with 10 or fewer claims to protect beneficiary privacy, resulting in underestimating the pooled costs and claims. (TIFF) [file pone.0198674.s002.tiff]
